# Supplementary material for: Effects of pentoxifylline in patients with chronic Chagas cardiomyopathy: A randomized, double-blind, controlled pilot trial
Source: PLoS Negl Trop Dis. 2025 Oct 9;19(10):e0013555. doi: 10.1371/journal.pntd.0013555 (PMC12558611; doi:10.1371/journal.pntd.0013555)
Supplement: Attachment — Submitted filename: Revision.docx [file pntd.0013555.s002.docx]

General comments:

I would like to thank the editors and authors for the opportunity to contribute by reading and commenting on this MS. The MS is very interesting, and I have tried to enhance my comments to the level of this contribution.

Chagas disease is a neglected disease and several aspects of its pathogenesis, diagnosis and therapy deserve much attention. In this parasitic disease, the time for etiological therapy is often lost due to the alleviated acute phase. The patient's fate is unclear and in the chronic phase the therapeutic regimen used only attenuates the clinical signs and symptoms. There is an urgent need for rational therapies, particularly considering the pathogenic mechanisms proposed to underly the most frequent form of Chagas disease, chronic cardiomyopathy. Based on the current corollary that pathogenesis of the cardiac form of Chagas disease is underlined by (i) the presence of scarce parasitism, (ii) low-grade local inflammation, (iii) systemic inflammatory profile and (iv) intrinsic cardiac tissue injuries, the proposal of a therapeutic approach focused on regulating the unbalanced immune response deserves much attention and careful interpretation of the results.

The here presented therapeutic proposal for CCC using pentoxifylline is based on a hypothesis supported by previous findings in non-infectious human cardiomyopathies and preclinical studies using mouse and hamster models of chronic Chagas cardiomyopathy. In the present study, the therapeutic regimen is the commonly used (pentoxifylline - 400 mg 3x/day), but the therapy duration (“prolonged”, “long-term”) is a point to be discussed (was it sufficient?). The parameters analyzed are very well chosen, the experimental design is adequate (baseline versus endpoint; proposed therapy versus placebo, double blinded analysis). However, the results are not well shown, and some information may be lost (showing data only in means +/- SD). Therefore, the MS cannot be accepted in its current format and requires major revision.

Lastly, improving quality of life is a very relevant issue for Chagas disease patients. I am glad it was considered in the present study.

Specific comments:

**Title:**

Line 1 - “Effects of prolonged…”. “Prolonged” is an imprecise adjective and should be avoided in the title. Particularly considering that 6 months of PTX administration regimen was used for non-infectious ischemic cardiomyopathy (Sliwa et al 2004) and it was not considered a long-term use. Further, the chronic Chagas cardiomyopathy flourishes after decades of parasitism and inflammatory lesions. Due to the nature of the infection and the individual differences, this reviewer should expect the need for a longer therapeutic regimen to observe more prominent results in Chagas disease. Please, consider removing this word of the title. Further, a support for the use of this therapy duration should be bring in the introduction section and this point should be considered in Discussion section.

**Abstract:**

Data presentation (means +/- SD) may put down relevant findings (particularly considering “individuals”) and may indicate that the sample size calculation was inappropriate. Unfortunately, this appears to be the case (see comments: Results).

Line 57: “Long-term” (here used as synonyms of prolonged) – should be considered for exclusion from this sentence.

**Introduction:**

This section is mostly well-written.

Line 73: “The chronic phase, which is symptomatic…” most patients with Chagas disease are asymptomatic. Even among patients with cardiac disease, many are asymptomatic, although EKG and/or ECHO abnormalities may be detected when patients are clinically evaluated. The entire sentence (lines 73–76) should be rephrased to avoid misleading readers.

Lines 82-86: This sentence brings a restrict and doubtful point of view. Please, rephase it and, at least, present a divergent point of view.

Kierszenbaum in his article “[Where do we stand on the autoimmunity hypothesis of Chagas disease?](https://pubmed.ncbi.nlm.nih.gov/16125464/) (Trends Parasitol. 2005 Nov;21(11):513-6. doi: 10.1016/j.pt.2005.08.013) assumed that this deleterious idea (here presented as “probably autoimmunity mechanisms” supported by a review of the group) has delayed research in Chagas disease. “The notion that autoimmunity underlies pathogenesis in Chagas disease has had a negative impact on efforts to develop effective chemotherapy and vaccines against *Trypanosoma cruzi* infection.” I invite the authors to read Kierszenbaum's article and to revisit the literature in depth in search of support for autoimmunity in Chagas disease.

The proposed use of PTX in Chagas disease may find support in data showing chronic cardiac injury enriched in TNF+ cells (Higuchi’s studies) and in the dysregulated “systemic inflammatory profile” found in sera of patients with heart disease (Nunes et al., 2022 and earlier by Ferreira et al., Perez-Fuentes et al., Perez et al., Dutra et al.), which is supported by findings in preclinical models showing high levels of inflammatory cytokines in plasma and cardiac tissue.

The choosing of cytokines as inflammatory mediators used as readouts for systemic inflammation should be better supported.

Line 120: Again, “the prolonged” should be supported. Why do the authors consider 6 months a long-term treatment?

**Methods and Population.**

Electrocardiograms are routine and the data should be presented as they can provide relevant information.

Lines 225-159: It is not my field of study, and the images and information are very good and help to follow the results. However, if standardized protocols were used for myocardial perfusion analysis (cited reference #28), why were Fig 1 and Fig 2 shown in Methods? Is there a need to have any specific analysis/interpretation for Chagas disease cardiopathy? If yes, please use it as the result. If not, please use it as supporting information.

Line 271: I could not find “Appendix 1”. If the questionnaire is available online in English version, please indicate link for access.

The study population was classified as NYHA I or II. Have you included patients with severe CCC (like C patients, according to the Guidelines by Marin-Neto et al, 2023). Why was LVEF the parameter (5% difference in 6 months?) used to calculate sample size? Please, explain it in the text and support it by reference.

Results

EKG are routine and the data should be presented as they can provide relevant information.

Table 1: Was the disequilibrium of male gender, hypertension, and NYHA Class II between the PLC versus PTX groups corrected?

Table 1. Patients are classified as NYHA I or II are using ACE inhibitors, β-blockers, diuretics, spironolactone. Continued throughout the study period? Any other medication? Hormones (particularly considering gender imbalance – and the effects of hormones on cytokine expression/levels)? Considering the parasitological origin of the disease, were they treated etiologically (benznidazole? other?)? Recent data support that prior treatment with antiparasitic medication (benznidazole) may influence TNF levels.

Table 2: The results of cytokine evaluation are interesting. The small number of patients per group may contribute to explaining p-values, and the analysis used (data are only shown as means+/- SD). Considering the common variations of cytokine levels in serum of Chagas disease patients (a quite individual disease that may be influenced by parasite diversity, period after infection, neuro-hormonal and immunological conditions of patients), data should be presented as individuals using graphs showing levels before and after treatment, at least for TNF, IL-6 and IL-10.

The endothelin-1 levels should be reviewed (it seems quite irreal = **ED-1 (pg/mL)** 4.37 ± 98 // 5.58 ± 15.2). Are they correct?

Lazzerini et al proposal of the contribution of heart inflammation in prolonged QTc syndrome, and the participation of EKG alterations found in Chagas disease patients, particularly prognostic value of QT interval (Salles et al. 2003), were the EKG analyzed in these groups of patients? Has PTX any impact on EKG parameters, particularly on QT/QTc interval?

Table 3: At baseline, pro-BNP levels are very dissimilar. PLC = 1207.1 ± 2343.5 versus PTX = 654.9 ± 1226.9. The final assessment also shows differences PLC = 2762.8 366 ± 8098.4 versus PTX = 508.4 ± 464.7. What should these differences be due to? Were all dosages performed at the same time and using the same kit/plate/reagents?

Line 377: do you mean Table 4? Line 412: do you mean Table 5?

Table 5: The importance of r myocardial perfusion deficits (defects?) justifies the presentation of data as individuals (graphs before and after treatment). I could not find the results of ischemic deficits before and after treatments.

Table 6. CF = functional capacity, CF should be FC; AF = physical aspects, AF should be PA / PhA

Considering the number of patients, the borderline p-values seen in some parameters analyzed, the biological aspects (when the gain of 5-10% of beneficial response in a 6-month interval may have importance for the patient's quality of life), I suggest that the authors take a multiparameter view of each group of patients in a principal component analysis (PCA). This analysis can add a more understandable view of the results. Furthermore, it can establish any possible correlation between variables.

Considering the high relevance of this study and the 5-year period after patient inclusion/therapy, is it possible to bring survival surveillance and/or record cardiovascular events of this small group of patients? This could provide relevant information.

Discussion

This section is mostly well-written and may gain with the revision of Results.

Lines 460-462 versus Line 494: I agree that IL-6 is an interesting cytokine to be evaluated and considered in the pathogenesis of CCC (particularly considering the type of analysis and graphs and, moreover, the multiparametric analysis I have suggested). However, these 2 sentences are not coherent. Please, revise it after the suggested analysis.

Line 492: It is not coherent. Table 2 shows p = 0.06 (“…TNF-α levels in the PTX-treated group (p=0.05)”). If you used different models of analysis, you should present them.

Line: 481-482: “Thus, the majority of the patients had mild cardiomyopathy, in early or intermediate stages of CCC, corresponding to stages B1, B2 and C of CCC progression”. However, according to The SBC Guideline on the Diagnosis and Treatment of Patients with Cardiomyopathy of Chagas Disease – 2023 (Marin-Neto et al. 2023)**,** the patients classified as C patients have severe form of CCC (Table 5.2: Structural cardiac disease; global systolic ventricular dysfunction; previous or current symptoms of HF), although they may be classified as NYHA class I, II, III or IV. This makes me wonder: 1- Are you comparing comparable groups of patients? (Table 1 shows more NYHA class I (maybe B1/B2?) in the PTX group); What is the percentage of C patients in each group? 2- Why did you adopt the NYHA classification if Brazil has a First (2011) and a Second (2023) Guidelines for CCC classification?

Lines 549-568: These are very interesting points. I am glad that you discussed preclinical models, which are often ignored in MS focused on clinical trials or clinical CCC. Preclinical models reproduce aspects of CCC (particularly those that are highly conserved across species). I appreciate the comment that Etanercept is not a good TNF blocker. Another preclinical study using a better TNF blocker (Infliximab) found very interesting results in a CCC model.

However, the duration of treatments (30 or 60 days in models) should not be considered to compare with the 6-month treatment used in the present study. The progression of CCC in models and humans is not comparable. In humans, CCC is a long-lasting and very heterogeneous disease, in contrast to the preclinical model. Even compared to any other human cardiomyopathies (ischemic, for example), CCC is a long-lasting process (probably with different pathogenic mechanisms, as parasite persistence) and is expected to require longer therapy, as you have considered in Discussion.

A Limitation section is required.
