# Supplementary material for: Effects of pentoxifylline in patients with chronic Chagas cardiomyopathy: A randomized, double-blind, controlled pilot trial
Source: PLoS Negl Trop Dis. 2025 Oct 9;19(10):e0013555. doi: 10.1371/journal.pntd.0013555 (PMC12558611; doi:10.1371/journal.pntd.0013555)
Supplement: Attachment — Submitted filename: Rebuttal Letter.doc [file pntd.0013555.s004.doc]

Ribeirao Preto, July 11^th^, 2025

To Dr. Shaden Kamhawi

Editor-in-Chief, Journal of PLOS Neglected Tropical Diseases

Dear Dr. Kamhawi,

We did appreciate the editorial revision and the opportunity to improve our manuscript entitled *“Effects of pentoxifylline in patients with chronic Chagas cardiomyopathy: a randomized, double-blind, controlled pilot trial”.*

All the considerations of the editorial board were addressed, and the respective answers are presented below.

**REVIEWERS' GENERAL COMMENTS:**

**Reviewer #1:**

1. “I suggest the authors to include the limitations and repercussion of not including the number of sample size planned.”

*We included comments about the limitations imposed by the reduced sample size.*

2. I suggest the authors to compare the baseline NT-ProBNP level between placebo and PTX group because they are quite diferents and comment this difference

*We performed the comparison, as suggested, but found no difference. This result was included in the manuscript. (page 18; Line 395)*

3. Please include references in the last paragraph of the topic - Effects of Pentoxifylline on NT-ProBNP

*The last paragraph refers to the other results of the present study and not the results of other studies in the literature. This paragraph was edited to clarify this point. (page 28; Line 604)*

**Reviewer #2:**

4. “The main weakness of the paper is the small sample size. Appropriate power calculation were performed but the assumptions used were likely too small. a 5% difference in LVEF is likely within the test-to-test variability of echocardiography.

In fact, it is actually smaller than the 6% variability the authors provide, which also seems smaller than I would expect and is not supported by a citation.”

*We chose a 5% difference in the LVEF between the groups as we considered this difference to be significant after an intervention. This was based on a previous study of our research group following patients with CCC (Hiss FC, Lascala TF, Maciel BC, Marin-Neto JA, Simões MV. Changes in myocardial perfusion correlate with deterioration of left ventricular systolic function in chronic Chagas' cardiomyopathy. JACC Cardiovasc Imaging. 2009 Feb;2(2):164-72.).*

5. “The randomization scheme needs to be described in more detail.”

*We added details regarding the randomization process in the new version of the manuscript. (page 8; Line 167)*

6. “Consider reporting interquartile range instead of total range.”

*We accepted the suggestion, and the non-Gaussian distribution variables were described as median and interquartile range. (page 13; Line 292)*

7. “Participants who died were excluded from analyses, but cause of death should be provided (if available) and discussed.”

*We had 2 deaths during the study, 1 death in each group. Unfortunately, the information about the causes of the deaths is not available. Those patients were not included in any analysis, as they did not undergo the post-treatment assessments.*

8. “While it is unlikely that they affected the outcome, especially since there were similar numbers in each group, including lost to follow up and dead participants in a sensitivity analysis would strengthen the paper. This is especially important given that the overall sample size was so small.”

*We fully agree that the loss to follow-up, including participants who died before the end of the study period, represents an important limitation, particularly given the small sample size. However, the outcomes assessed in our study were defined as changes from baseline to the re-evaluation. For participants without a re-evaluation, it was therefore not possible to determine these outcomes or to conduct a sensitivity analysis incorporating their data. Nonetheless, we have included a statement acknowledging this limitation in the discussion section of the manuscript.*

9. “Prevalence of hypertension and diabetes were much higher in the placebo group than in the control group, which likely biased results since it does not appear that

these comorbidities were adjusted for when comparing outcomes. Even though this was an RCT, these differences probably should be adjusted for as they almost certainly affect LVEF trajectories.”

*We thank the reviewer for raising this important point. The prevalence of hypertension was indeed significantly lower in the treatment group compared to the placebo group. However, no differences were observed in systolic or diastolic blood pressure between groups, suggesting that hypertension was effectively managed in both. For this reason, we believe the risk of bias arising from this imbalance is minimal. Nonetheless, we have acknowledged this potential limitation in the discussion section. Finally, although the proportion of participants with diabetes was higher in the placebo group, this difference did not reach statistical significance.*

10. “The authors do not describe how they defined systemic hypertension. Was this self reported? the mean BP for each group was in the normal range despite a hypertension prevalence of 89% in the placebo group.”

*The presence of systemic hypertension was based on self-reported data. The BP was probably normal because the patients were on medications to control their BP.*

11. “The observed changes in cytokine levels are not surprising since pentoxifylline is an anti-inflammatory. In this study, it is not really possible to connect these changes with any clinical outcomes.”

*We observed a trend towards the reduction of the pro-inflammatory cytokine levels with pentoxifylline use. As the other cardiac function and myocardial perfusion variables presented no significant change between the groups, we could not establish any relationship between those results. The results are discussed considering this perspective.*

12. “They admit that the short duration of follow up (6 months) may have been too short to detect a clinically significant decline in EF, which is more likely to occur on a scale of years.”

*We agree with the comment.*

13. “Line 412: I think they are referring to table 5 not table 4.

*This mistake was corrected*.

15. “Figure 4 and 5 are not really necessary and could be deleted or moved to a supplement.”

*We accepted the suggestions and deleted figures 4 and 5.*

16. “line 483: I think they are referring to table 6 not table 5.”

*This mistake was corrected.*

**Reviewer #3:**

17. “A limitation section is necessary.”

*This mistake was corrected.*

**SPECIFIC COMMENTS**

18. “Line 1 - “Effects of prolonged…”. “Prolonged” is an imprecise adjective and should be avoided in the title. Particularly considering that 6 months of PTX administration regimen was used for non-infectious ischemic cardiomyopathy (Sliwa et al 2004) and it was not considered a long-term use. Further, the chronic Chagas cardiomyopathy flourishes after decades of parasitism and inflammatory lesions. Due to the nature of the infection and the individual differences, this reviewer should expect the need for a longer therapeutic regimen to observe more prominent results in Chagas disease. Please, consider removing this word of the title. Further, a support for the use of this therapy duration should be bring in the introduction section and this point should be considered in Discussion section”

*We edited the title to exclude the term “prolonged”, as suggested. We included a comment about the treatment duration in the methods section. In the discussion, we comment on the influence of treatment duration on the results.*

***Abstract:***

19. “Data presentation (means +/- SD) may put down relevant findings (particularly considering “individuals”) and may indicate that the sample size calculation was inappropriate. Unfortunately, this appears to be the case (see comments: Results).”

*We agree with the comments, and these limitations were presented in the respective session.*

21. “Line 57: “Long-term” (here used as synonyms of prolonged) – should be considered for exclusion from this sentence.”

*The “long-term” was excluded from the sentence.*

***Introduction:***

22. “Line 73: “The chronic phase, which is symptomatic…” most patients with Chagas disease are asymptomatic. Even among patients with cardiac disease, many are asymptomatic, although EKG and/or ECHO abnormalities may be detected when patients are clinically evaluated. The entire sentence (lines 73–76) should be rephrased to avoid misleading readers.”

*The paragraph was edited. (page 4, Line:83)*

23. “Lines 82-86: This sentence brings a restrict and doubtful point of view. Please, rephase it and, at least, present a divergent point of view. Kierszenbaum in his article “[Where do we stand on the autoimmunity hypothesis of Chagas disease?](https://pubmed.ncbi.nlm.nih.gov/16125464/) (Trends Parasitol. 2005 Nov;21(11):513-6. doi: 10.1016/j.pt.2005.08.013) assumed that this deleterious idea (here presented as “probably autoimmunity mechanisms” supported by a review of the group) has delayed research in Chagas disease. “The notion that autoimmunity underlies pathogenesis in Chagas disease has had a negative impact on efforts to develop effective chemotherapy and vaccines against Trypanosoma cruzi infection.” I invite the authors to read Kierszenbaum's article and to revisit the literature in depth in search of support for autoimmunity in Chagas disease. The proposed use of PTX in Chagas disease may find support in data showing chronic cardiac injury enriched in TNF+ cells (Higuchi’s studies) and in the dysregulated “systemic inflammatory profile” found in sera of patients with heart disease (Nunes et al., 2022 and earlier by Ferreira et al., Perez-Fuentes et al., Perez et al., Dutra et al.), which is supported by findings in preclinical models showing high levels of inflammatory cytokines in plasma and cardiac tissue. The choosing of cytokines as inflammatory mediators used as readouts for systemic inflammation should be better supported.”

*We partly agree with the first remark above, and rephrased the sentence, that now reads: “Evidence from both animal and human studies indicates that two main pathogenic mechanisms may contribute to the development of chronic Chagas cardiomyopathy (CCC): persistent, low-grade but virtually incessant inflammation directly elicited by tissue parasitism and also, although with less evidence basis,*

*exaggerated inflammatory injury probably due to autoimmune mechanisms.” (Page 5; Line 95)*

*In regard to Kierszenbaum's classical paper, we actually quoted it in various articles our group published over the years. On the other hand, we thank the Reviewer and took the opportunity to re-read it along with the other related papers as suggested above. However, for the sake of preserving the main scope of our research, we respectfully would rather avoid dwelling into an extended use of PTX in Chagas patients. In fact, we plan to revisit that line of investigation in the future, taking into account the nice suggestions advanced by the Reviewer.*

24. “Line 120: Again, “the prolonged” should be supported. Why do the authors consider 6 months a long-term treatment?”

*The term prolonged was excluded.*

***Methods and Population***

25. “Electrocardiograms are routine and the data should be presented as they can provide relevant information.”

*The results of the EKG were included in the manuscript. (Page 12; Line 270)*

26. “Lines 225-159: It is not my field of study, and the images and information are very good and help to follow the results. However, if standardized protocols were used for myocardial perfusion analysis (cited reference #28), why were Fig 1 and Fig 2 shown in Methods? Is there a need to have any specific analysis/interpretation for Chagas disease cardiopathy? If yes, please use it as the result. If not, please use it as supporting information.”

*We think the quantitative methods used to evaluate the myocardial perfusion changes were not so common and deserve more details and illustration in the methods session*.

27. “Line 271: I could not find “Appendix 1”. If the questionnaire is available online in English version, please indicate link for access.”

*The mention of Appendix 1 was a mistake that was corrected in the new version of the manuscript.*

28. “The study population was classified as NYHA I or II. Have you included patients with severe CCC (like C patients, according to the Guidelines by Marin-Neto et al, 2023). Why was LVEF the parameter (5% difference in 6 months?) used to calculate sample size? Please, explain it in the text and support it by reference.”

*Information about the LVEF parameter to calculate the sample size was included in the text. (Page 13, Line 313)*

***Results***

29. “Table 1: Was the disequilibrium of male gender, hypertension, and NYHA Class II between the PLC versus PTX groups corrected?”

*We thank the reviewer for pointing this out. The prevalence of hypertension was indeed significantly lower in the treatment group compared to the placebo group. However, no diferences were observed in systolic or diastolic blood pressure between groups, suggesting that hypertension was effectively managed in both groups. For this reason, we believe the risk of bias arising from this imbalance is minimal. Nonetheless, we have acknowledged this potential limitation in the discussion section. Finally, although the proportion of men was higher in the treatment group and the proportion of participants classified as NYHA functional class II was higher in the placebo group, these 7iferences did not reach statistical significance.*

30. “Table 1. Patients are classified as NYHA I or II are using ACE inhibitors, β-blockers, diuretics, spironolactone. Continued throughout the study period? Any other medication? Hormones (particularly considering gender imbalance – and the effects of hormones on cytokine expression/levels)? Considering the parasitological origin of the disease, were they treated etiologically (benznidazole? Other?)? Recent data support that prior treatment with antiparasitic medication (benznidazole) may influence TNF levels.”

*The medications for heart failure/myocardial dysfunction were continued throughout the study period, even if the patients were asymptomatic, as recommended by the current guidelines. No other medications were registered. The patients were not treated with benznidazole.*

31. “Table 2: The results of cytokine evaluation are interesting. The small number of patients per group may contribute to explaining p-values, and the analysis used (data are only shown as means+/- SD). Considering the common variations of cytokine levels in serum of Chagas disease patients (a quite individual disease that may be influenced by parasite diversity, period after infection, neuro-hormonal and immunological conditions of patients), data should be presented as individuals using graphs showing levels before and after treatment, at least for TNF, IL-6 and IL-10.”

*We thank the reviewer for the comment and have added the suggested graphs. (Figure 4 – page 17; Line 380)*

32. “The endothelin-1 levels should be reviewed (it seems quite irreal = **ED-1 (pg/mL)** 4.37 ± 98 // 5.58 ± 15.2). Are they correct?”

*The results are correct and were confirmed.*

33. “Lazzerini et al proposal of the contribution of heart inflammation in prolonged QTc syndrome, and the participation of EKG alterations found in Chagas disease patients, particularly prognostic value of QT interval (Salles et al. 2003), were the EKG analyzed in these groups of patients? Has PTX any impact on EKG parameters, particularly on QT/QTc interval?”

*The analysis of the EKG, including the QTc results, is now included in the manuscript. (Page 23; Line 484)*

34. “Table 3: At baseline, pro-BNP levels are very dissimilar. PLC = 1207.1 ± 2343.5 versus PTX = 654.9 ± 1226.9. The final assessment also shows differences PLC = 2762.8 366 ± 8098.4 versus PTX = 508.4 ± 464.7. What should these differences be due to? Were all dosages performed at the same time and using the same kit/plate/reagents?”

*The dosages were performed at the same time with the same kit. The differences were attributed to chance.*

35. *“*Line 377: do you mean Table 4? Line 412: do you mean Table 5?”

*This mistake was corrected.*

36. “Table 5: The importance of myocardial perfusion deficits (defects?) justifies the presentation of data as individuals (graphs before and after treatment). I could not find the results of ischemic deficits before and after treatments.”

*Ischemic defects are described as reversible ones. We thank the reviewer for the comment and have added the suggested graphs. (Figure 5 - Page 20; Line 449)*

37. “Table 6. CF = functional capacity, CF should be FC; AF = physical aspects, AF should be PA / PhA”

*These mistakes were corrected.*

38. “Considering the number of patients, the borderline p-values seen in some parameters analyzed, the biological aspects (when the gain of 5-10% of beneficial response in a 6-month interval may have importance for the patient's quality of life), I suggest that the authors take a multiparameter view of each group of patients in a principal component analysis (PCA). This analysis can add a more understandable view of the results. Furthermore, it can establish any possible correlation between variables.”

*We performed a PCA analysis and included the results. (Page 24; Line 514)*

39. “Considering the high relevance of this study and the 5-year period after patient inclusion/therapy, is it possible to bring survival surveillance and/or record cardiovascular events of this small group of patients? This could provide relevant information.”

*We agree that this could be a relevant information, but we didn’t consider perform this analysis because the treatment period was relatively short.*

***Discussion***

40. “Lines 460-462 versus Line 494: I agree that IL-6 is an interesting cytokine to be evaluated and considered in the pathogenesis of CCC (particularly considering the type of analysis and graphs and, moreover, the multiparametric analysis I have suggested). However, these 2 sentences are not coherent. Please, revise it after the suggested analysis.”

*We revised the sentences.*

41. “Line 492: It is not coherent. Table 2 shows p = 0.06 (“…TNF-α levels in the PTX-treated group (p=0.05)”). If you used different models of analysis, you should present them.”

*We corrected this mistake.*

42. “Line: 481-482: “Thus, the majority of the patients had mild cardiomyopathy, in early or intermediate stages of CCC, corresponding to stages B1, B2 and C of CCC progression”. However, according to The SBC Guideline on the Diagnosis and Treatment of Patients with Cardiomyopathy of Chagas Disease – 2023 (Marin-Neto et al. 2023)**,** the patients classified as C patients have severe form of CCC (Table 5.2: Structural cardiac disease; global systolic ventricular dysfunction; previous or current symptoms of HF), although they may be classified as NYHA class I, II, III

or IV. This makes me wonder: 1- Are you comparing comparable groups of patients? (Table 1 shows more NYHA class I (maybe B1/B2?) in the PTX group); What is the percentage of C patients in each group? 2- Why did you adopt the NYHA classification if Brazil has a First (2011) and a Second (2023) Guidelines for CCC classification?”

*We adopted the NYHA as this classification is based on symptom severity, a relevant parameter for disease assessment. The evolutive stages are also important and were mentioned in the text. We edited the text to make this point clearer.*

43. “Lines 549-568: These are very interesting points. I am glad that you discussed preclinical models, which are often ignored in MS focused on clinical trials or clinical CCC. Preclinical models reproduce aspects of CCC (particularly those that are highly conserved across species). I appreciate the comment that Etanercept is not a good TNF blocker. Another preclinical study using a better TNF blocker (Infliximab) found very interesting results in a CCC model. However, the duration of treatments (30 or 60 days in models) should not be considered to compare with the 6-month treatment used in the present study. The progression of CCC in models and humans is not comparable. In humans, CCC is a long-lasting and very heterogeneous disease, in contrast to the preclinical model. Even compared to any other human cardiomyopathies (ischemic, for example), CCC is a long-lasting process (probably with different pathogenic mechanisms, as parasite persistence) and is expected to require longer therapy, as you have considered in Discussion.”

*We thank the reviewer for their relevant comments.*

44. “A Limitation section is required.”

*We agree and have included a limitations section.*
